# An assessment of oil palm plantation aboveground biomass stocks on tropical peat using destructive and non-destructive methods

**DOI:** 10.1038/s41598-020-58982-9

**Published:** 2020-02-10

**Authors:** Kennedy Lewis, Elisa Rumpang, Lip Khoon Kho, Jon McCalmont, Yit Arn Teh, Angela Gallego-Sala, Timothy Charles Hill

**Affiliations:** 1 Geography, College of Life and Environmental Sciences, University of Exeter, Streatham Campus, Rennes Drive, Exeter, EX4 4RJ UK; 2 Tropical Peat Research Institute, Biological Research Division, Malaysian Palm Oil Board, 6, Persiaran Institusi, Bandar Baru Bangi, 43000 Kajang, Selangor Malaysia; 3 School of Natural and Environmental Science, Newcastle University, Drummond Building, Newcastle-upon-Tyne, NE1 7RU UK

**Keywords:** Agroecology, Ecology, Carbon cycle, Environmental sciences, Environmental impact

## Abstract

The recent expansion of oil palm (OP, *Elaeis guineensis*) plantations into tropical forest peatlands has resulted in ecosystem carbon emissions. However, estimates of net carbon flux from biomass changes require accurate estimates of the above ground biomass (AGB) accumulation rate of OP on peat. We quantify the AGB stocks of an OP plantation on drained peat in Malaysia from 3 to 12 years after planting using destructive harvests supported by non-destructive surveys of a further 902 palms. Peat specific allometric equations for palm (R^2^ = 0.92) and frond biomass are developed and contrasted to existing allometries for OP on mineral soils. Allometries are used to upscale AGB estimates to the plantation block-level. Aboveground biomass stocks on peat accumulated at ~6.39 ± 1.12 Mg ha^−1^ per year in the first 12 years after planting, increasing to ~7.99 ± 0.95 Mg ha^−1^ yr^−1^ when a ‘perfect’ plantation was modelled. High inter-palm and inter-block AGB variability was observed in mature classes as a result of variations in palm leaning and mortality. Validation of the allometries defined and expansion of non-destructive inventories across alternative plantations and age classes on peat would further strengthen our understanding of peat OP AGB accumulation rates.

## Introduction

Global demand for palm oil has risen such that the land area supporting oil palm (OP, *Elaeis guineensis*) plantations has increased to ~25 Mha globally; making OP the 12^th^ largest edible crop by land area^1^. The rapid expansion of OP in Insular Southeast Asia during the last quarter decade has resulted in the conversion of 3.1 Mha of tropical peatlands^2^. The carbon emissions from the oxidation of soil organic matter following the conversion of peat swamp forest to OP are relatively well known, yet the net carbon emission of peat swamp forest conversion to OP across the life of a plantation remains poorly constrained^3–6^. In part, uncertainty is attributed to a scarcity of literature which addresses the rate at which OP on peat accumulates carbon in biomass over time^6–10^. The majority of OP standing biomass is stored as aboveground biomass (AGB) constituting 84% of biomass stocks, with the reminder (16%) stored as belowground biomass (BGB); consequently, efforts here focus primarily on AGB quantification^11–13^.

Recent efforts to quantify the AGB stocks of forests and plantations have increasingly used remote sensing techniques^14,15^. However, remote sensing estimates ultimately rely on direct ground-based measurement of AGB stocks either for calibration or validation^15,16^. Forest and plantation vegetation is destructively harvested to obtain the vegetation dry-weight (DW) and infer biomass carbon stocks (~47.4% of dry biomass)^17,18^. These destructive measurements are essential but are costly in terms both of time and resources; allometric equations which relate AGB stocks to non-destructive or semi-destructive measurements of vegetation structural characteristics are therefore invaluable^18,19^. Destructive and non-destructive AGB stock estimates are common for OP on mineral soils but are almost entirely absent for OP on peat^6,8,10,20^. Furthermore, much of the literature and allometries are contained within ‘*grey’* literature. The lack of published direct ground-based estimates of AGB for OP on peat is also a major limitation for remotely sensed estimates of OP AGB and in carbon bookkeeping models^6,21,22^.

**Figure 1:**
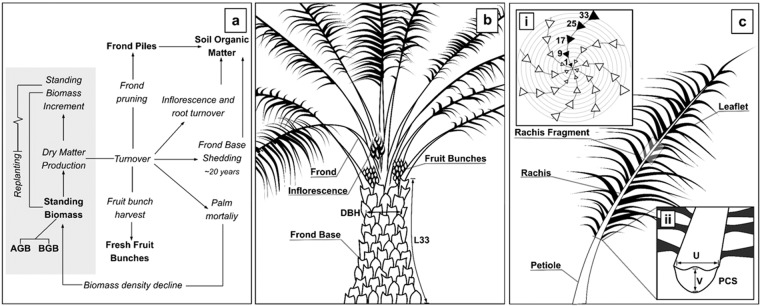
Oil palm AGB components, turnover and measurement. (**a**) Biomass turnover and stocks across a 25-year planting cycle. (**b**) An upright Young Mature oil palm with DBH (measured at 1.3 m excluding frond bases) and trunk length to the frond ranked 33 (L33) indicated. (**c**) Labelled frond diagram, (ci) indicates frond rank numbering and crown phyllotaxis (after Aholoukpè *et al*., 2013), (cii) demonstrates PCS (petiole cross sectional area) measurement where PCS = U × V, a rachis fragment is taken from the rachis midpoint.

OPs are typically managed for a planting cycle of ~25 years after which profitability reduces and the next cropping rotation is initiated^23^. However, during each growing cycle only a proportion of the biomass produced is retained by the palm to augment its existing biomass, the remainder is lost as a result of the natural and managed turnover of fruit, inflorescences, fronds and frond bases (Fig. 1a)^10,23,24^. Fruit bunches develop in the axil of each frond and are harvested cyclically. Fronds emerge at a rate of 20–25 fronds per year and are progressively pruned before being plied on the plantation floor during harvesting rounds^23,25^. Frond bases; which are left adhering to trunk subsequent to pruning accumulate during the early to middle years of the planting cycle and are typically shed ~12 years after planting^23^. The single growing apex of OPs, absence of secondary stem thickening once mature and regular phyllotaxis of fronds within the palm crown mean they are well suited to dry weight quantification and allometric development (Fig. 1b,c)^26,27^. On mineral soils allometric equations have been produced to monitor each palm AGB component in order to accurately equate biomass stocks and turnover spatially and over time (Table 1). However, many OP AGB assessments state biomass values without information pertaining to planting density and local environment and are subject to uncertainties associated with a lack of standardised methods (Supplementary Table S1)^8,9^. Models of OP biomass stock accumulation on mineral soils have also been developed and have been incorporated into large scale LUC carbon flux and bookkeeping models^8,21,28–30^.

**Table 1:** Existing allometric equations for the estimation of OP component dry weight (kg) and OP AGB accumulation models for OP on mineral soils.

No	Component	Equation	Source	Note
**Allometries Tested**				
1	Frond DW	$D{W}_{Frond}=0.102\times PCS+0.21$	Corley *et al*., 1971	—
2	Frond DW	$$\begin{array}{c}D{W}_{Frond}=\alpha +\,\beta \times PCS\\ \alpha =-0.0076+0.0394\times YAP\\ \beta =0.0284+0.0101\times YAP\end{array}$$	Henson (1993): in Hanson and Dolmat 2003	Palms YAP ≤ 6
3	Rachis DW	$D{W}_{Rachis}=1.133\times \frac{D{W}_{Frag}}{{L}_{Frag}}\times {L}_{Rachis}$	Aholoukpè *et al*., 2013	—
	Frond DW	$D{W}_{Frond}=1.147+2.135\times D{W}_{Rachis}$		
4	Trunk DW	$D{W}_{Trunk}={T}_{Vol}\,\times \rho =\rho (\pi {r}^{2}\times {L}_{Trunk})$	Corley *et al*., 1971	Trunk biomass without frond bases
	Trunk Density	$\rho =0.0076\times YAP+0.083$		
**Biomass Accumulation Models**				
M1	Standing Biomass (Mg ha^−1^)	$$\begin{array}{lll}SB & =\, & -0.00020823\times YA{P}^{4}\times 0.000153744\times YA{P}^{3}\\ & & -0.011636\times YA{P}^{2}+7.3219\times YAP-6.3934\end{array}$$	Henson, 2003	Standing biomass, adjusted to AGB (Morel *et al*., 2011).
M2	Aboveground Biomass (Mg ha^−1^)	$AGB=18.95\times YA{P}^{0.5}$	Germer and Sauerborn, 2006	—
M3	Aboveground Biomass (Mg ha^−1^)	$AGB=1.526(5.97\times YA{P}^{0.62})$	Carlson *et al*., 2012	Model adjusted to carbon to AGB

Where DW_Frond_ is frond dry weight (kg), PCS is the petiole cross sectional area (cm), DW_Rachis_ is rachis dry weight (kg), DW_Frag_ is rachis fragment dry weight (kg), L_Frag_ is rachis fragment length (m), L_Rachis_ is rachis length (m), DW_Trunk_ is trunk dry weight, T_Height_ is trunk height (m), DW_Palm_ is palm dry weight (kg), T_Vol_ is trunk volume (m^3^), DBH is the diameter at breast height (m) and YAP is years after planting.

OP plantations on peat are markedly different to those on mineral soils with potential impacts on AGB stock estimations. Following the clearance of forest biomass, peatlands are drained to an optimum water table depth 0.4–0.6 m from the peat surface to allow cultivation^31–33^. Peat bulk density is increased to ~0.20 g cm^−1^ by mechanical compaction using heavy machinery, often including the compaction of residual forest material into the peat^31,34,35^. This increases the load-bearing capacity of peat soils and improves the anchorage of OPs which allocate a relatively small proportion of total biomass to belowground root systems^11,32–34^. Following this initial compaction further peat subsidence occurs as a result of peat shrinkage, consolidation and decomposition following drainage^36^. This subsidence, when combined with poor root anchorage, frequently results in individual palms leaning at an angle to the ground. As leaning becomes more severe roots become exposed and vulnerable to desiccation and breakage which can result in the palms falling over entirely, the likelihood of this increases as palms mature with associated gains in trunk and crown biomass^32^. This has become a serious limiting factor for OP performance on peat and will likely have detrimental effects on AGB stocks as plant density per area is reduced due to palm mortality (Fig. 1a)^10,32,33^. Initial palm planting densities ore optimised for maximum fresh fruit bunch (FFB) yield across the life of the plantation, higher densities are therefore adopted for less favourable soils^24^. In contrast to OP on mineral soils, optimal planting densities on peat range from 160 to 200 palms per hectare (110–148 palms per hectare on mineral soils)^10,24,33^.

In this study, we quantify the AGB (dry-weight) of OPs on deep peat in Sarawak, Malaysia. Destructive harvests of nine palms split amongst three age classes (IM: immature, YM: young-mature and M: mature) are supported with non-destructive measurements and surveys of a further 902 palms. Harvest data is used to develop new allometric equations for palm and component AGB. Non-destructive measurements are then used to upscale the destructive harvests to the plantation block level. We develop models of AGB accumulation rates on peat to inform existing OP AGB growth and carbon balance models. Finally, a meta-analysis of existing OP allometries for palms on both peat and mineral soils is performed and the results contrasted with data and allometries developed as part of this study.

## Results

### OP biomass distribution in immature, young-mature and mature palms

**Figure 2:**
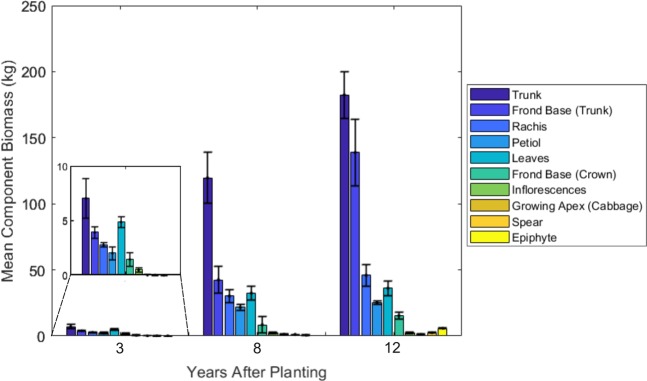
Mean AGB component dry weights (kg) for immature, young mature and mature OPs. Error bars indicate standard error. Frond Base (Crown) is the remaining frond base left in the crown subsequent to live frond removal (see methods).

Of the palms destructively harvested, one mature palm was mildly leaning (Supplementary Table S2). As expected, the palm trunk makes the largest contribution (33 to 46%) to the total palm dry weight (DW_palm_), particularly in the YM and M classes (Fig. 2). Frond base biomass also constitutes a large proportion of the overall biomass (13 to 32%), again particularly in the older age classes (Fig. 2, Table 2). Palm trunks retained all frond bases in all palms harvested. In immature palms, fronds make up a larger proportion of overall biomass.

**Table 2:** Mean AGB component dry weights (kg) for immature, young mature and mature OPs (standard error indicated).

YAP	Stem			Frond				Spear	Cabbage	Total (All)
	Trunk	Frond Base	Total	Rachis	Petiole	Leaflet	Total			
3	7.0 ± 1.8	3.9 ± 0.5	11.0	2.8 ± 0.2	2.0 ± 0.9	4.9 ± 0.5	9.7	0.3 ± 0.1	0.3 ± 0.1	21.3 ± 5.9
8	111.8 ± 19.3	42.5 ± 10.2	154.3	30.2 ± 4.9	21.3 ± 2.6	32.3 ± 5.1	83.8	1.2 ± 0.1	1.3 ± 0.6	240.6 ± 15.3
12	182.4 ± 17.6	138.8 ± 25.2	321.2	45.7 ± 8.1	25.0 ± 1.4	36.1 ± 5.7	106.9	2.4 ± 0.4	1.3 ± 0.5	431.8 ± 90.1

**Figure 3:**
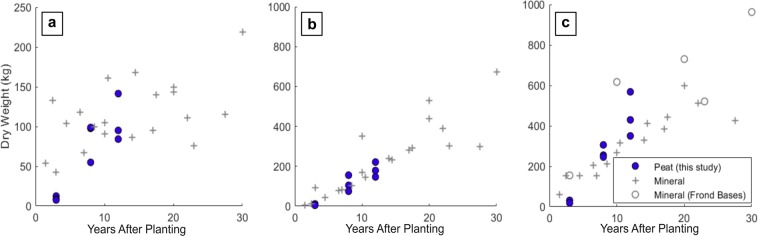
Dry weights of OP components (kg). Dry weights where quantified using destructive harvests including total frond biomass per palm (**a**), palm trunk biomass (**b**) and palm biomass (excluding fruit and epiphytes) (**c**). Per palm DWs of OP AGB components on mineral soils are taken from Corley *et al*. (1971), Khalid *et al*., (1999), Rees and Tinker (1963) and Syahrinudin (2005). Frond base biomass is included in palm (total) where reported ((**c**) - grey open circle).

Contrasting palm trunk and total frond dry weight for each age class to those on mineral soils revealed no differences (Fig. 3). However, accessible data was scarce on both mineral and peat soils.

### Allometric estimation of palm and frond component biomass

Harvest data was used to validate existing allometric equations and develop equations for Malaysian OP on deep peat (Tables 1 and 3).

**Table 3:** Allometric equations for the estimation of OP component dry weight (kg) and OP AGB accumulation models for OP on peat soils.

No	Component	Equation	Note
**Derived Allometries**			
5	Frond DW	$D{W}_{Frond}=0.060\times PCS+0.217$	Frond DW estimation using the petiole cross sectional area of a pruned frond.
6	Rachis DW	$D{W}_{Rachis}=1.126\times \frac{D{W}_{Frag}}{{L}_{Frag}}\times {L}_{Rachis}$	Frond DW estimation using the DW of a rachis fragment taken from a pruned frond.
	Frond DW	$D{W}_{Frond}=0.176+2.267\times D{W}_{Rachis}$	
7	Frond DW	$D{W}_{Frond}=0.562\times {L}_{Rachis}-0.767$	Frond DW estimation using rachis length.
8	Palm DW	$$\begin{array}{c}D{W}_{Palm}=12.87+560.8\times {T}_{Vol}\\ {T}_{Vol}={(\pi \times 0.5\times DBH)}^{2}\times {L}_{Trunk}\end{array}$$	Palm DW estimation derived from non-destructive trunk volume measurement. DBH measured excluding frond bases.
**Derived Biomass Accumulation Models**			
P1	Aboveground Biomass (Mg ha^−1^)	$AGB=6.389\times YAP-17.59$	AGB accumulation on peat – observed plantation biomass.
P2	Aboveground Biomass (Mg ha^−1^)	$AGB=7.992\times YAP-26.29$	AGB accumulation on peat - ‘perfect plantation’ model. All palms are modelled as live and standing.

Allometric equations are derived from destructive harvest data at the study site. Where DW_Frond_ is frond dry weight (kg), PCS is the petiole cross sectional area (cm), DW_Rachis_ is rachis dry weight (kg), DW_Frag_ is rachis fragment dry weight (kg), L_Frag_ is rachis fragment length (m), L_Rachis_ is rachis length (m), DW_Trunk_ is trunk dry weight, T_Height_ is trunk height (m), DW_Palm_ is palm dry weight (kg), T_Vol_ is trunk volume (m^3^), DBH is the diameter at breast height (m) and YAP is years after planting.

### Frond DW estimation

Existing allometric equations estimating frond dry weight (DW_frond_) using the petiole cross sectional area (PCS) (Equation (1) and (2)) and rachis linear density (RLD) (Equation (3)) were tested. The petiole cross sectional area is the sectional area at the junction of the petiole and rachis (at the point of insertion of the lowest leaflet) (Fig. 1c). The rachis linear density is derived from the dry weight of a rachis fragment and is used to predict rachis dry weight (DW_Rachis_) and infer DW_Frond_.

All existing allometric equations tested overestimated frond dry weight (Supplementary Fig. S1). Frond DW estimation using the petiole cross sectional area (Equation (1)) overestimated DW_frond_ by ~56% for young mature and mature palms and ~119% for immature palms. However, using to Equation 2 to estimate DW_frond_ from the PCS for palms < 6 years after planting improved estimation in the immature age class, overestimating frond dry weight by only 21%. Estimation using rachis linear density (Equation (3)) resulted in an overestimation of ~61% for young mature and mature palms and ~300% for immature palms. Rachis dry weight was however well predicted from rachis linear density (Equation (3), Supplementary Fig. S2). Further allometries referred to in Corley and Tinker (2016) both over and underestimated DW_Frond_ (Supplementary Fig. S3).

**Figure 4:**
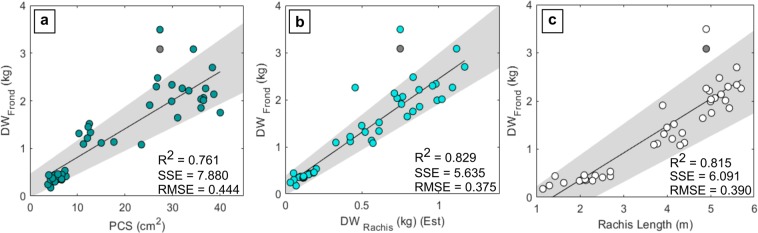
Linear relationship between frond structural characteristics and Frond DW (DW_Frond_). DW_Frond_ is compared to the petiole cross sectional area (PCS) ((**a**) - equation (5)), rachis dry weight (DW_Rachis_) derived from rachis linear density ((**b**) – equation (6)) and rachis length ((**c**) – equation (7)). A total of 45 fronds were sampled, fronds ranked 1, 9, 17, 25 and 33 were sampled for each of the nine destructively harvested palms. 95% confidence interval of fit indicated in grey; consistent outliers indicated as a black closed circle.

Allometric relationships for DW_frond_ estimation on deep peat were then defined. Frond dry weight in each age class was lower than reported for palms on mineral soils but was more consistent with those sampled by Henson and Dolmat (2003) from OPs on peat (Supplementary Fig. S4). Leaflets in immature palms made a larger contribution to overall frond dry weight when compared to the mature age classes (Supplementary Fig. S5), equations were adjusted to include all palm ages sampled. Rachis linear density was a marginally better predictor of DW_Frond_ (R^2^ = 0.83), when compared to the petiole cross section (R^2^ = 0.76) once adjusted to harvested fronds (Fig. 4). However, estimation of DW_Frond_ using the petiole cross sectional area was considered more practical in the field. Rachis length was also used to predict DW_Frond_ to a similar degree of accuracy (R^2^ = 0.81).

### Palm DW estimation

**Figure 5:**
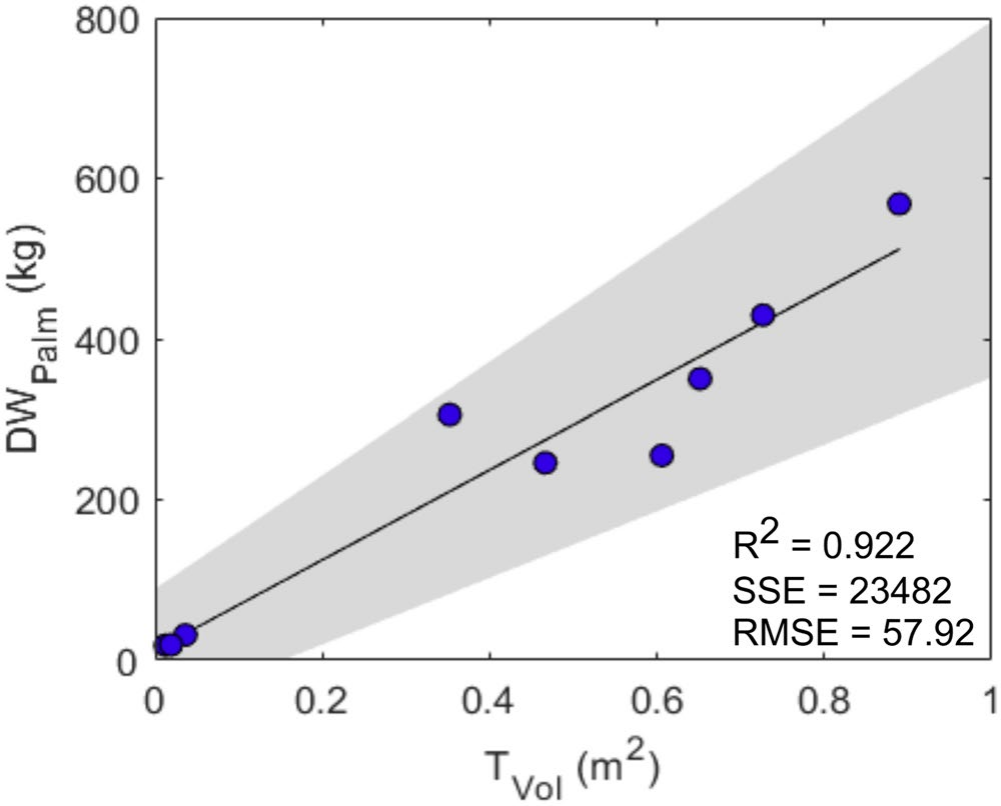
Linear relationship between palm trunk volume (T_Vol_) and palm dry weight (DW_Palm_) for the nine destructively sampled OPs. 95% confidence interval of fit indicated in grey (Table 2, equation (8)).

The palm trunk makes the greatest proportional contribution to overall palm biomass (Fig. 2). Equation (4) underestimated trunk dry weight by 32% in YM and M palms (frond bases not included). Total palm DW (DW_palm_) is estimated using trunk height (height to frond 33) in existing allometries (Supplementary Table S3). Whilst trunk length was found to be a good estimator of DW_palm_ (R^2^ = 0.88), the use of trunk volume was marginally more effective for the palms sampled (R^2^ = 0.92) (Equation (8)) (Fig. 5). A model was developed to predict DW_palm_ excluding frond bases to simulate frond base shedding, however R^2^ = 0.52, potentially due to a small sample size (n = 6) and the highly variable contribution of frond bases to the overall DW_palm_ of palms sampled.

### Upscaling biomass to the plantation block scale

**Figure 6:**
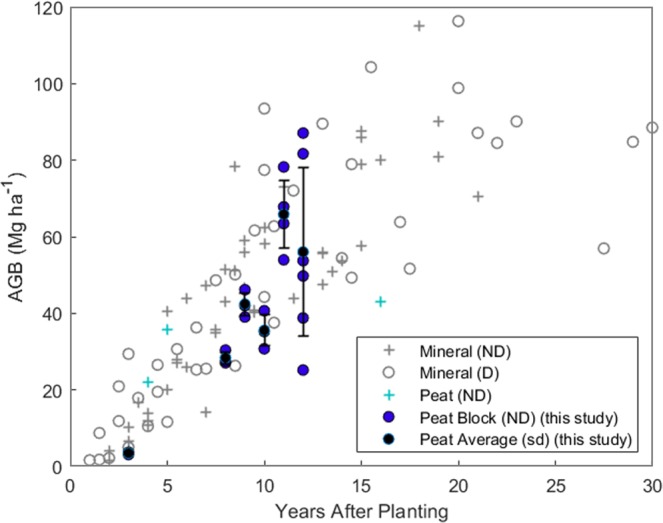
Oil palm block-level cumulative AGB stock (Mg ha^−1^) for peat (blue markers) and mineral soils (grey markers). OP aboveground biomass stocks on mineral soils (Table S1) were obtained using destructive (D) and non-destructive (ND) methods and are presented in addition to existing values for OP on peat. Existing data for non-destructive mineral estimates (grey+) and destructive mineral (open grey circle) and non-destructive peat (green +). Block AGB stocks at the study site are included (closed blue circle) and the plantation mean for each YAP plotted (closed black circle), standard deviation indicated (Black error bars).

Non-destructive measurements were combined with the allometric equations defined for OP on peat to assess biomass stocks at the plantation block level. Equation (8) was used to estimate the biomass stock of live palms in 22 0.25 ha plots in plantation blocks at various stages of maturity (Fig. 6). This confirmed a large variation in biomass stocks in the more mature plots with a mean AGB of 65.9 ± 8.7 Mg ha^−1^ 11 years after planting and 56.04 ± 12.0 Mg ha^−1^ after 12 years (Fig. 6). When a ‘perfect’ plantation on peat is modelled, disregarding fallen, missing and re-planted palms (which represented 13% of palms in plots > 8 YAP) aboveground biomass stocks accumulated at ~7.99 ± 0.95 Mg ha^−1^ yr^−1^ in the first 12 years after planting. However, this is reduced to ~6.39 ± 1.12 Mg ha^−1^ per year considering all 22 assessed plantation blocks when palm mortality and replacement is taken into account. Mild and severely leaning palms made up 17% of live palms in plots > 8 YAP, however, inter-plot variation within age classes across the plantation was high.

**Figure 7:**
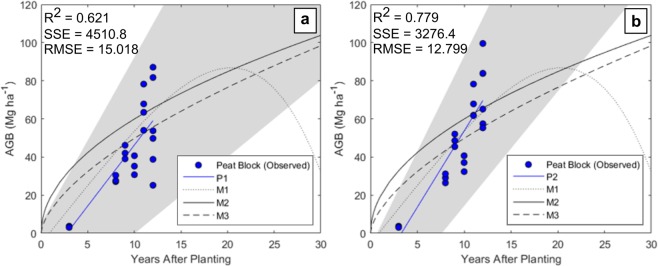
AGB accumulation models (Mg ha^−1^) for oil palm on deep peat from 3 to 12 YAP. (**a**) Models observed OP accumulation at the Sabaju and Sebungan OP estate complex (Model P1). (**b**) Models a ‘perfect’ plantation on peat modelling all palms as live, present and standing (Model P2). 95% confidence intervals of both fits indicated in grey. Existing AGB accumulation models for OP on mineral soils (YAP 0–30) are plotted (Models 1, 2 and 3, Table 1).

Aboveground biomass stocks at the study site were compared to assessments of OP AGB on mineral soils in addition to comparison with AGB accumulation models (Fig. 6). Only 3 accessible assessments of OP AGB stocks on peat soils where available (Fig. 6). At the time of survey there were no planting blocks aged > 12 YAP at the study site. Henson (2003), Model M1, assumes an AGB reduction ~18 years after planting due to frond base shedding. In contrast, Models M2 and M3 do not indicate this reduction (Fig. 7). Peat OP AGB at the Sabaju and Sebungan Estates appears consistent with OP on mineral soils. However, in mature blocks where palm falling and missing palms were common AGB stocks were notably lower than modelled OP growth (Figs. 6 and 7).

## Discussion

### Dry weight distribution of OP on peat

The dry weight of OPs in three age classes was quantified using destructive harvests. As palms transitioned from youth to maturity trunk length and dry weight increased, this was also accompanied by an increase in frond base biomass relative to the total palm dry weight. Studies that destructively harvest frond bases to quantify biomass are few when compared with other AGB components^12,30,37,38^. This is likely due to the practical difficulties associated with frond base removal^37^. It is often also unclear whether non-destructive OP biomass assessments that quantify plantation biomass stocks using allometries have included the dry weight contribution of adhering frond bases^8,9^. Henson *et al*. (2012) found total frond base dry biomass per palm to be 10.8, 62.8 and 56.0 kg, 3, 10 and 13 years after planting in Papua New Guinea (with 94.6% of frond bases adhering to the trunk 13 YAP). Frond bases made an even greater contribution to overall palm biomass in this study, particularly in mature palms (Table 2). A review of studies quantifying frond base biomass highlights the high variation in palm frond base dry weight when compared to both palm age and trunk biomass^37^. Despite this variation, frond bases make a large contribution to the overall AGB of OP plantations in the young mature and mature age classes and will become a large carbon source following shedding before the end of the plantation planting cycle as frond base litter decomposes^37,39^.

The biomass of a single mature frond grown on peat was consistently lower than on mineral soils in all age classes when compared to pooled frond DWs for palms on mineral soils (Supplementary Fig. S4)^40^. Studies have also found the rate of frond emergence to reduce significantly as planting density is increased^10,41^. Taking into account the higher planting density of OP on peat, it is therefore surprising that there was no observable difference between total per palm frond biomass on mineral and peat soils (Fig. 3a). The acidity, low nutrient content and poor fertiliser retention of managed tropical peat soils is likely to result in reduced vegetative dry matter production and biomass accumulation when compared to OP on mineral soils^42^. In addition to this, palms at higher densities are subjected to increased competition for light thus reducing the dry matter production per palm^23^. Despite these expectations, our study revealed no notable differences in palm, trunk or frond biomass between mineral and peatland plantations. However, the lack of available literature which documents DW_Palm_, DW_Trunk_ and the total frond biomass for individual palms on mineral soils and the small sample size of palms on peat in this study makes it difficult to identify significant differences in palm and component biomass. Difference may however be detectable with a larger sample size. To confound this, palms on mineral soils have been sampled using non-standardized methodologies and are influenced by differences in genotype, eco-region and plantation management^8,11,12,26,30^.

### Allometric equations for OP component DW on peat

This study defined allometric relationships for OP and OP component dry biomass on drained tropical peats. Allometries produced here for the estimation of frond dry weight incorporate fronds of various ranks from multiple age classes. Here, the frond rachis linear density and petiole cross sectional area were both effective predictors of DW_Frond_ (R^2^ = 0.82, R^2^ = 0.76). In contrast to Corley and Tinker (1971) (Equation (1)), Aholoukpè *et al*. (2013) found frond biomass to be poorly predicted using the PCS in YM and M palms (R^2^ = 0.22) but found rachis linear density to be a better predictor (R^2^ = 0.62). However, the increased effort required to measure rachis linear density from the dry weight of a rachis fragment in the field is perhaps not justified by the marginally stronger relationship between rachis linear density and DW_Frond_ when compared to using the petiole cross sectional area in this study. An allometry was defined relating trunk volume to the total palm biomass (Equation (8)). To take into account the structural variation of OP on peat T_Vol_ was modelled as a cylinder the length of the trunk to F33, measuring along the inner curve of the trunk for leaning palms^32^.

### Application of existing allometries to peat OP

Frond biomass for palms on peat was overestimated by the majority of existing allometric equations tested (derived using palms on mineral soils), most notably in the immature age class. This overestimation of young palm DW_Frond_ is also acknowledged by Henson (1993) and a large improvement was observed when applying Equation (2), which is adjusted for use on young palm fronds. Equation (3) has yet to be validated for young palm fronds and whilst DW_rachis_ was well estimated for all age classes, adjustment is needed before it can be used for young palm DW_Frond_ prediction on peatlands^43^.

In the mature age classes, trunk biomass was underestimated by ~32% when using Equation (4), much greater underestimation of ~10% acknowledged by Morel *et al*. (2011)^44^ when using this allometry. Corley *et al*. (1971) model the trunk (without frond bases) as a cylinder with a constant diameter with wood density estimated according to palm age. Aholoukpè *et al*. (2018)^45^ attempted reduce the uncertainty introduced though these assumptions by modelling the true inverted cone shape of the stem and incorporating the linear density of the trunk. However, this assumes an upright palm and hence is often not applicable to OP on peat due to high incidence of palm leaning^32^.

Here palm dry weight was best predicted using trunk volume. Thenkabail *et al*. (2004) relate DW_Palm_ to trunk height in Benin; the resulting allometry greatly underestimated DW_Palm_ in mature and young mature palms in this study resulting in a mean underestimation of 72%. However, no palms with a trunk height > 1.95 m were incorporated into the initial model. Dewi *et al*. (2009)^46^ produced a similar allometry for OP on mineral soils in Indonesia which can be used more successfully with a mean underestimation of only 16% when applied here to OP on peat (Supplementary Table S3).

### Plantation block-level AGB

The allometries developed using destructive sampling were combined with non-destructive palm structural measurements and frond pruning to upscale biomass stock estimates to the planation block level. Trunk DBH remained consistent across the age classes (YAP > 8) whilst trunk length increased with age in standing palms (Supplementary Fig. S6). In ‘successful’ blocks, per hectare AGB was similar to that observed on mineral soils (Fig. 6). Vegetative dry matter production and standing biomass per hectare increases with planting density as observed in studies on both peat and mineral soils, disregarding fruit bunch biomass^10,47^. The higher planting density of palms on unfavourable peat soils likely contributes to the high per hectare AGB stocks in plots where leaning is infrequent or mild with relatively few fallen palms^24,33^. However, there is a large variation in plot per hectare AGB within age classes and in plots with a high incidence of leaning and fallen palms AGB was greatly reduced. Here, mild and severely leaning palms made up 17% of live palms in plots > 8 YAP with an additional 13% of OPs fallen, missing or replaced. However, inter-plot variation within age classes across the plantation was high (Fig. 6). Census of the incidence of palm leaning were carried out at 6-month intervals in an experimental OP block on deep peat in Sarawak^48^. After 12 years 50.3% of palms were mildly leaning and 2.8% had fallen or were severely leaning, this worsened to 55.5 and 6.9% in uncompacted plots^48^. Dolmat *et al*. (1995) found leaning incidences of 44.2 (compacted) and 71.9% (uncompacted) in Perak.

As a result of the recent rise in OP expansion across tropical peats combined with efforts to increase peat OP sustainability, research increasingly focuses on the optimisation of peat OP growth and fruit bunch yields^23,32,33^. Prior to conversion, site and soil surveys are of high importance as the position on the peat dome, peat composition, maturity and depth have all been found to have an impact on conversion success, palm growth and yield potential^20,35^. Peat compaction to increase bulk density prior to conversion and the thorough removal of woody debris from forest clearance is important to improve palm anchorage, whilst maintenance of a consistent water table increases palm rooting depth potential^32,33,35,49,50^. Once palms have reached maturity and leaning has commenced regular pruning to reduce canopy biomass and prevent toppling in addition to soil mounding roots after exposure both aid in reducing palm falling and limit AGB and yield reductions^51^.

### Limitations and further work

In addition to the limitations highlighted, further uncertainties arise from the focus of this study on a single plantation. We observed a high variation in palm structural characteristics and plot biomass stocks within mature age classes in a single well managed industrial OP estate. Therefore, the actual variation of monoculture OP plantation AGB stocks on peat across Sarawak, Malaysia and Insular Southeast Asia is likely to be greater considering differences in plantation management and leaning, peat properties and ecoregions.

The sample size of destructively harvested palms is small (n = 9), with few mature palms and no palms > 12 YAP harvested. Similar studies which destructively harvest palms on mineral soils to quantify DW_Palm_ include between 3 to 10 palms sampled for each palm age and span from 1.5 to 33 years after planting (Supplementary Table S3)^11,12,26,30,52^. Small sample sizes are common in destructive biomass assessments due to costly sampling procedures (particularly in older, larger palms) and results are therefore vulnerable to the influence of variation between individual palms^9^. We acknowledge the need to extend the temporal scope of the chronosequence here to include mature palms > 12 YAP as AGB stocks after this point are uncertain. This could inform growth models for OP on peat beyond ~18 YAP where existing models of OP AGB accumulation vary (Fig. 7)^3,21,28,53^. Continuing the chronosequence would also permit the averaging of biomass stocks across the life of a plantation on peat, aiding in the comparison of biomass stocks with alternative land cover types for LUC flux modelling and carbon accounting^6,54^. Here all palm mortality and replacement has been attributed to palm leaning in the plots considered, however the spread of pests (particularly termites on peat soils) and diseases such as *G. boninense* basal stem or trunk rot are also frequently the cause of palm failure and replanting^55,56^. Despite this, the plantation studied here is in its first planting cycle and with no instances of *G. boninense* observed^23^.

Finally, all allometric relationships defined here would benefit from validation to test their success on OP on drained peats, including mature palms as well as their possible application in alternative ecoregions and at different planting densities^17^.

## Conclusion

The recent rapid expansion of OP plantations across managed tropical peatlands is known to result in net carbon emissions. However, the emissions associated with this land use change across the life of a plantation remain poorly constrained as aboveground biomass accumulation rates on peat are uncertain due to a lack of both destructive and non-destructive AGB quantifications.

Here, we produce peat OP specific allometries for the estimation of both palm and frond dry weight and use these allometries to upscale AGB estimates to the plantation block level. This revealed a high variability in aboveground biomass stocks across a plantation in the mature age classes. Increasing non-destructive inventories on peat will not only improve AGB accumulation models but could also inform *in-situ* remote sensing efforts to quantify AGB stocks. Validating the allometries produced by expanding destructive harvests across different plantations on peat in addition to including older palms in harvests and plot inventories would further strengthen our understanding of peat OP AGB stock changes over time.

## Methodology

### Study site

Measurements were carried out at the Sebungan and Sabaju Oil Palm Estate Complex, Sarawak, Malaysia (3.19˚N 113.43˚E). The industrial OP plantation has an area of ~10,200 ha. The site receives ~3075 mm rainfall per year with an average temperature of 27.2 °C. Meteorology was recorded at 1-minute intervals on a Sutron XLite 9210B datalogger (Sterling, Virginia, US). Air Temperature was measured at 1 m using a Vaisala HMP155 (Vaisala, Helsinki, Finland). Precipitation was measured at 6 m, i.e. above the canopy, using a Texas Electronics TR525M (Dallas, Texas, US).

The plantation is low lying, soil surveys indicate a majority composition of lowland organic deposits with an underlying marine clay mineral layer (84.8%). Very deep peat (>3m thick) covers the majority of the plantation; 42.2% has highly decomposed sapric surface (0–0.5 m) and subsurface (0.5–1.5 m) tiers. A further 42.6% is comprised of a partially decomposed sapric surface tier (0–0.6 m) and hemic subsurface tier (0.5–1 m). Both deposit types contain partially decomposed wood between 0.5–1 m.

Prior to conversion the site was covered in logged mixed peat swamp forest (PSF). Land preparation included the removal of remaining large trees and vegetation, the establishment of a drainage system and peat compaction using heavy machinery^31^. OPs are planted at a density of 160 palms per hectare and at the time of measurement ranged from 3 to 12 years after planting (YAP).

### Destructive harvests

#### Palm selection and sampling

Three palms were destructively harvested from each age class: 3 (Immature – I), 8 (Young Mature – YM) and 12 (Mature - M) years after planting. Palms were selected at random at least 50 m from the block edge, all were selected in different planting blocks, GPS coordinates were recorded (Supplementary Table S5). Severely leaning or recovered palms were not considered for destructive harvests. Prior to felling, non-destructive measurements of palm structural characteristics were taken.

#### Destructive measurements

All fresh weights (FW) (kg) were measured and recorded at the felling site as close to the time of felling as possible, with particular attention paid to leaflets. Samples were promptly transferred to the lab oven to avoid capturing decomposition in DW measurements.

Fronds. Fronds were removed from the palm crown as close as possible to the base of the frond using a harvesting sickle (Fig. 1c). Fronds were counted and any petiole remaining in the crown subsequent to frond removal was harvested and classified as ‘crown frond base’.

Using the frond rankings of Thomas *et al*., 1969, fronds 1, 9, 17, 25 and 33 were subsampled for allometric validation and development (Fig. 1ci). The petiole cross sectional area, rachis length and the fresh weights of the frond rachis, petiole and leaflets were recorded (Fig. 1c). Petiole cross sectional area was measured using callipers at the junction of rachis and petiole (the point of insertion of the lowest leaflet) and was modelled as a rectangle (*PCS* = *U* × *V*, Fig. 1cii)^11^. A 0.15 m fragment was removed from the midpoint of the rachis and petiole, a subsample of leaflets was also removed. All remaining fronds were split into components (rachis, petiole and leaflets) and their total fresh weight recorded.

Trunk and frond bases. All epiphytes were removed from the palm trunk, the FW of epiphytes was recorded, and a subsample taken. All frond bases were removed from the palm trunk and a disk ~0.2 m thick was removed from the trunk midpoint. This disk was weighed, and two perpendicular disk diameters recorded, a sector (~1/8^th^ of the disk) was removed and the fresh weight recorded, and the sector returned to the labs for DW analysis. The palm trunk (without frond bases) was then weighed using suspended scales at the felling site or at the plantation weighbridge. Subsequent to removal, the total FW of all frond bases was recorded, a subsample of 3 frond bases was then returned to the labs.

Inflorescences, fruit, spear and cabbage. The total FW of all inflorescences and fruit bunches and the palm spear and cabbage (growing apex) was recorded at the felling site before removing 3 subsamples per component for DW analysis. Fruit bunch fresh and dry weights where not included in any further analysis due to variation in palm harvesting cycles.

#### Laboratory analysis

Palm component subsamples were dried at 105 °c until a constant mass was reached, component moisture contents were then calculated for each sample.

### Non-destructive surveys and frond pruning

#### Plot selection and sampling

Non-destructive survey plots were selected at random across the plantation complex (with a minimum of 3 plots selected for each age class). 22 plots with an area of 0.25 ha were surveyed. Plots were 3, 8, 9, 10, 11 and 12 YAP and were in independent planting blocks, GPS coordinates were recorded at plot corners. The YAP of each plot was checked against planting blocking maps, plots were established away from block edges (Supplementary Table S5).

#### Leaning categorisation

The condition of each palm with the 0.25 ha plot was recorded. Palms were categorised as upright, mildly leaning, severely leaning, recovered, fallen (dead/alive), missing or replanted (see Supplementary Table S4). The direction of lean was also recorded.

#### Non-destructive measurement and pruning

Each 0.25 ha plot contained approximately 40 palms, palms were numbered, and structural measurements taken for 10 randomly selected palms. The canopy height was recorded. Trunk length was measured along the trunk to frond 33 or the most mature frond, for leaning palms the trunk length was measured along the trunk inner curve (Fig. 1b, Supplementary Fig. S7). Trunk diameter at breast height (DBH, 1.3 m) was measured using callipers so as not to include frond bases, for palms < 1.3 m in height the diameter was taken at the trunk midpoint. Frond 33 was pruned from the canopy of the corresponding palm; rachis length was recorded, and petiole cross sectional area was measured using callipers.

### Meta-analysis and allometry validation

#### OP biomass stock estimates

All accessible literature publishing per hectare standing biomass (SB) and AGB stocks for OP on both peat and mineral soils using destructive and non-destructive methods was collected. Values were adjusted to AGB (Mg ha^−1^), carbon contents were assumed to be 47.4% of dry biomass^18^. Where SB was reported AGB was assumed to be 84% of total SB based on assessments of belowground biomass (BGB) on mineral soils conducted by Corley and Tinker, 1971 and Khalid *et al*.^12,13^ (Root biomass = 16.1 +/−5.3% of overall SB in palms 1.5–27.5 YAP).

#### Allometric equations

Allometries for estimating palm component biomass derived using the destructive harvest of OP on mineral soils were collected and validated. Existing equations in the main section of the text (Table 1) are defined in peer reviewed literature, additional allometries are listed in the supplementary material.

## Supplementary information

Supplementary information.
