# Supplementary material for: An assessment of oil palm plantation aboveground biomass stocks on tropical peat using destructive and non-destructive methods
Source: Sci Rep. 2020 Feb 10;10:2230. doi: 10.1038/s41598-020-58982-9 (PMC7010673; doi:10.1038/s41598-020-58982-9)
Supplement: Supplementary file 1 — Supplementary information. [file 41598_2020_58982_MOESM1_ESM.pdf]

An assessment of oil palm plantation aboveground biomass stocks on tropical peat using destructive and non-destructive methods

Kennedy Lewis[1], Elisa Rumpang[2], Lip Khoon Kho[2], Jon McCalmont[1], Yit Arn Teh[3], Angela Gallego-Sala[1], Timothy Charles Hill[1]

[1] College of Life and Environmental Science, University of Exeter, Streatham Campus, Rennes Drive. Exeter, EX4 4RJ. UK

[2] Tropical Peat Research Institute, Biological Research Division, Malaysian Palm Oil Board, 6, Persiaran Institusi, Bandar Baru Bangi, 43000 Kajang, Selangor, Malaysia

[3] School of Natural and Environmental Science, Newcastle University, Drummond Building, Newcastle-upon-Tyne, NE1 7RU UK

Corresponding Autor: Kennedy Lewis (contact: kl378@exeter.ac.uk)

Supplementary Material

Source	Method	Location/Region	Planting Density	Soil	Note
Henson and Dolmat (2003)[10]	ND	Peninsular Malaysia	160	Peat	
Melling et al (2007)[57]	ND	Sarawak, Malaysia	-	Peat	
Breure (1982)[58]	ND	Papua New Guinea	-	Mineral	
Breure (1988)[59]	ND	Papua New Guinea	-	Mineral	
Dufrene (1989)[60]	ND	Ivory Coast	-	Mineral	
Henson (unpublished, 1993-95)[40]	ND	Selangor, Malaysia	-	Mineral	
Kwan (1994)[41]	ND	Sabah, Malaysia	143	Mineral	
Henson (1995)[61]	ND	Selangor, Malaysia	-	Mineral	
Lamade and Setiyo (1996)[62]	ND	Sumatra, Indonesia	-	Mineral	
Henson (1998)[63]	ND	Selangor, Malaysia	-	Mineral	
Palm et al (1999)[64]	ND	Cameroon	-	Mineral	
Tjitrosemito and Mawardi (2000)[65]	ND	Indonesia			
Banabas (2002)[66]	ND	Papua New Guinea	130	Mineral	
Henson (2007)[67]	ND	Kedah, Malaysia	-	Mineral	
Morel et al (2011)[44]	ND	Sabah, Malaysia	-	Mineral	
Rees and Tinker (1963)[26]	D	Nigeria	-		Destructive harvest: 7 to 22 YAP, 3 repetitions per age class
Ng et al. (1968)[68]	D	Peninsular Malaysia	-	Mineral	
Corley et al (1971)[11]	D	Peninsular Malaysia	148	Mineral	Destructive harvest: 1.5 to 27.5 YAP, 38repetitions per age class
Khalid et al. (1999)[12,13]	D	Peninsular Malaysia	-	Mineral	Destructive harvest: 23 YAP, 10 repetitions
Thenkabail et al. (2004)[46]	D	Benin	-	Mineral	Destructive harvest: Trunk heights of 0.28 to 1.95 m, 7 palms samples (YAP unknown)
Syahrinudin (2005)[30]	D	Sumatra, Indonesia	-	Mineral	Destructive harvest: 3 to 33 YAP, 3 repetitions per age class
Legros et al. (2006)[69]	D	East Kalimantan, Indonesia	-	Mineral	
Koh et al. (2019)[70]	D, ND	Sarawak, Malaysia	-	Mineral	Destructive harvest: 21 YAP, 10 repetitions

Table S1: Oil palm plot AGB per hectare. *Source material for Figure 6. Outline of studies assessing OP aboveground biomass stocks on mineral soils and peat soils using destructive (D) and non-destructive (ND) methods, planting densities included where possible.*

Sample No	YAP	No of Fronds	Trunk DBH (m) *	Trunk Length (m)**	Lean Category***
1	12	49	0.50	4.53	M
2	12	40	0.48	3.60	U
3	12	35	0.50	3.70	U
4	8	40	0.69	1.62	U
5	8	34	0.63	1.13	U
6	8	40	0.64	1.45	U
7	3	35	0.45	0.23	-
8	3	38	0.27	0.22	-
9	3	41	0.37	0.18	-

Table S2: Characteristics oil palms destructively harvested

** Trunk DBH measured at breast height (1.30m) using callipers to exclude frond bases, trunk diameter was measured at the trunk midpoint where trunk heights were < 1.3m.*

*** Trunk length was measured to the frond ranked 33 (L33). Where palms were leaning, the trunk length along the inner curve of the palm trunk was recorded.*

**** Leaning categories: M = Mildly leaning, U = Upright.*

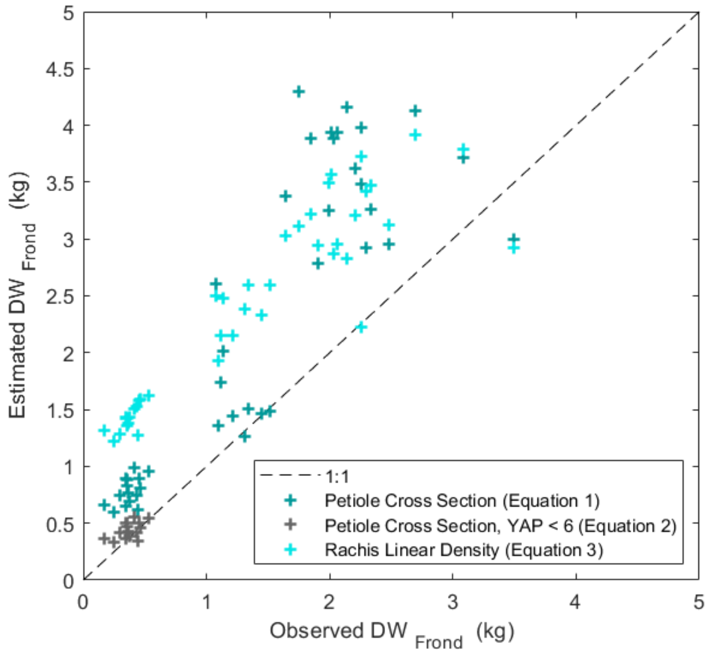

***Figure S1: Frond DW predicted using existing equations vs observed** frond DW (1:1 line indicated). The equations tested use the petiole cross section (Equation1, Corley et al, 1971[11] and Equation 2, Henson, 1993[10]) and the rachis linear density (Equation 3, Aholoukpè et al, 2013[43]) to estimate the DW of a single frond.*

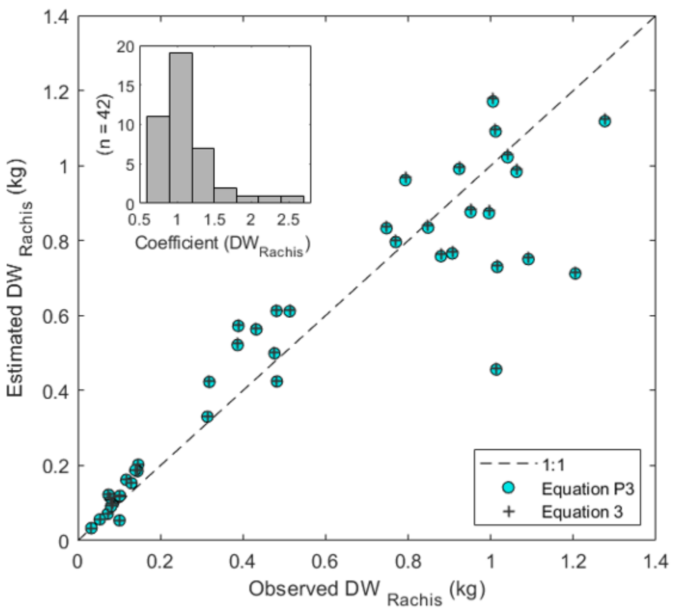

***Figure S2: Rachis dry weight (DW_{Rachis}) is estimated from the dry linear density of a rachis fragment. Rachis** DW predicted using an existing equation (Equation 3) and an equation derived for peat (Equation P3) are plotted against the observed rachis DW (1:1 line indicated). The distribution of coefficients accounting for the non-constant sectional area of the rachis for each frond are shown (Equation P3, top left).*

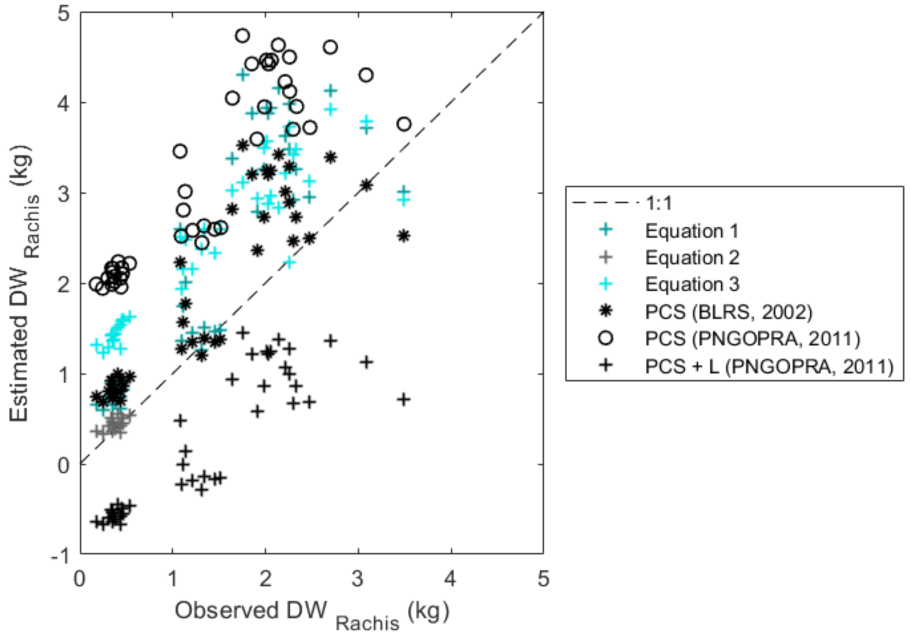

***Figure S3: Frond DW predicted using existing equations vs the observed frond DW (1:1 line indicated).** Equations tested use the petiole cross section (PCS) and the petiole cross section when combined with frond length (PCS + L) to estimate the DW of a single frond. Allometries recorded in Corley and Tinker, 2016[71,72].*

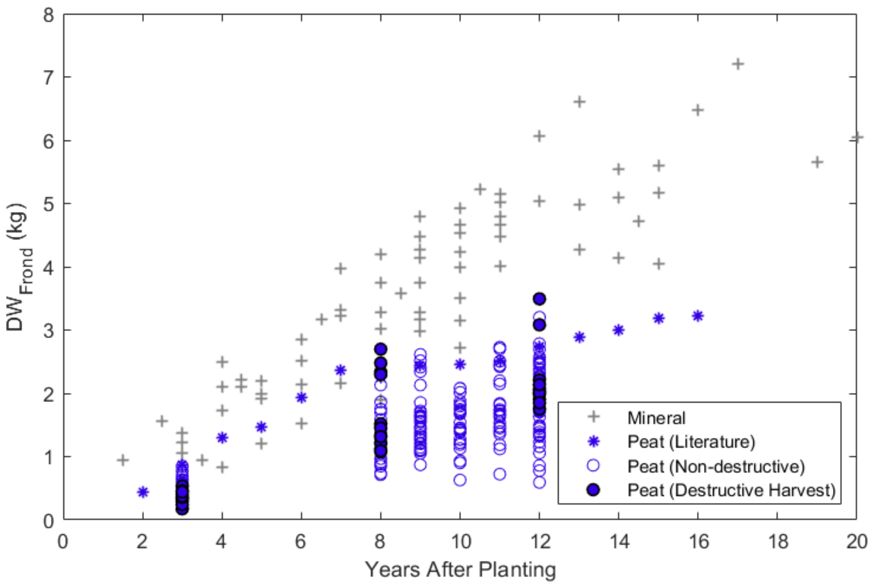

Figure S4: Single frond dry weights on mineral and peat soils. *Dry weight of fronds sampled in the non-destructive plot survey (Peat (Non-destructive)) are calculated using Equation P1, destructively harvested fronds are also included. Adapted from Henson 2005[29], including fronds on peat soils (Henson and Dolmat, 2003).*

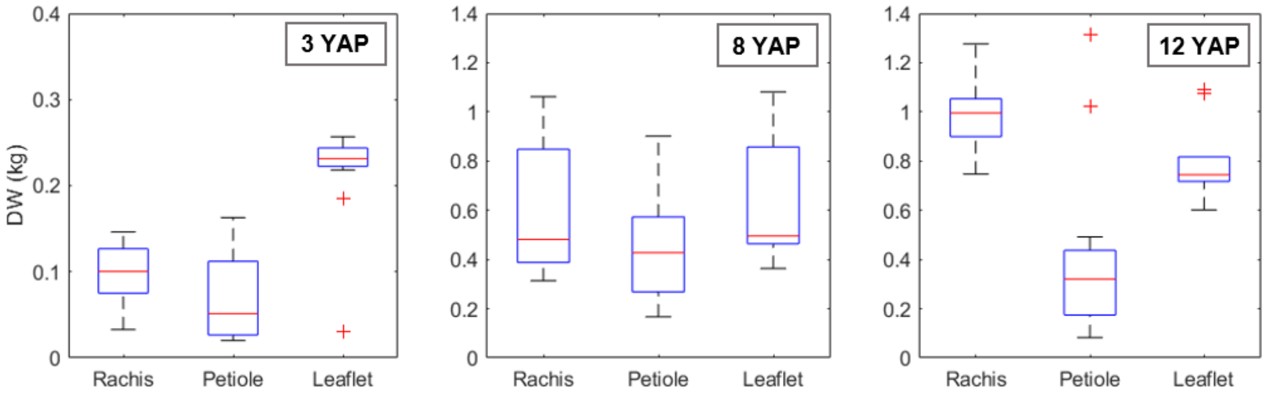

Figure S5: Frond component dry weight distribution in immature, young-mature and mature palms. Frond component dry with distribution of single fronds ranked 1, 9,17, 25 and 33 (rachis, petiole and leaflet) in immature (3 YAP), young-mature (8 YAP) and mature (12 YAP) palms. Outliers indicated in red.

No	Component	Equation	Reference	Note
S1	Palm DW	$DW_{Palm} = (0.0976 \times T_{Height} + 0.0706) \times 1000$	Dewi et al, 2009[47]	~0.5 > T_{height} > 9 (m)<br>Derived from semi-destructive methods<br>(R^2 = 0.7342)<br>Location: Indonesia
S2	Palm DW	$DW_{Palm} = 37.47T_{Height} + 3.6334$	Thenkabail et al, 2004[46]	N = 8<br>0.28 > T_{height} > 1.95 (m)<br>Location: Benin

Table S3: Existing allometric equations for estimating total oil palm dry weight (kg). *Where DW_{Palm} is palm dry weight and T_{Height} is trunk height to frond 33 (m).*

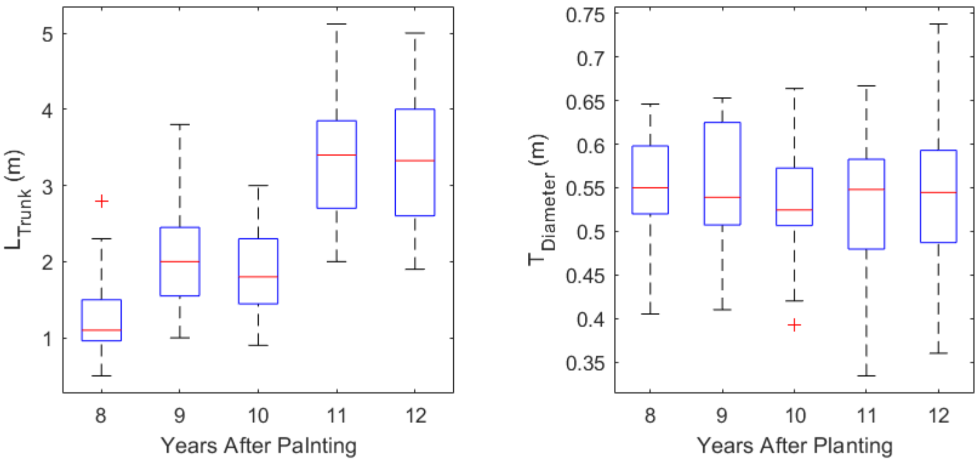

Figure S6: Trunk length (left) and DBH (right) (m) as measured in non-destructive surveys. *Data pooled for all plots of the same age. Outliers indicated in red.*

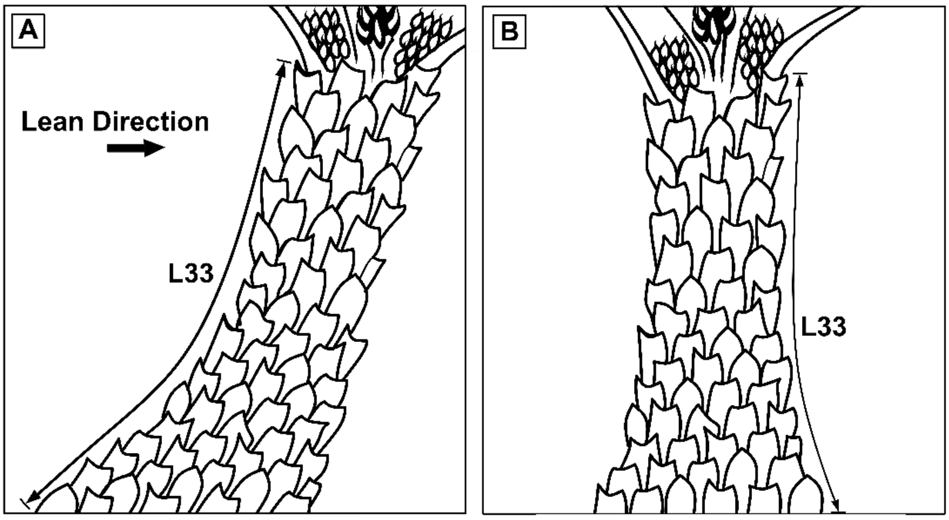

***Figure S7: Oil palm leaning and length measurement**. Diagram A shows a mildly leaning palm mature palm, Diagram B an upright palm. Trunk length (L33) is measured along the inner curve of the trunk parallel to the lean direction in mildly leaning palms.*

**Leaning Category**	
Upright	Upright
Mild	Leaning at < 45° from the vertical
Severe	Leaning at > 45° from the vertical
Recovered	Leaning palms returning upright state
Fallen (Alive)	Fallen live palm (parallel to the peat), partially rooted
Fallen (Dead)	Fallen dead palm (parallel to the peat), uprooted
Replanted	Immature palm, notably younger than the block age (refill palm following palm mortality)
Missing	Missing palm in planting grid

Table S4: Categorisation of Oil Palm Leaning on Tropical Peats

Plot Id	Lat (N)	Long (E)	YAP	Measurement
**1**	3.1773	113.3729	12	D, ND
**2**	3.1744	113.3697	12	D, ND
**3**	3.1705	113.3711	12	D, ND
**4**	3.1640	113.4187	8	D, ND
**5**	3.1622	113.4180	8	D, ND
**6**	3.1628	113.4162	8	D, ND
**7**	3.1609	113.4207	3	D, ND
**8**	3.1594	113.4207	3	D, ND
**9**	3.1604	113.4179	3	D, ND
**10**	3.1658	113.3524	9	ND
**11**	3.1884	113.4631	9	ND
**12**	3.1879	113.4612	9	ND
**13**	3.1846	113.4593	9	ND
**14**	3.2333	113.4792	10	ND
**15**	3.2328	113.4803	10	ND
**16**	3.2267	113.4723	10	ND
**17**	3.2267	113.5069	11	ND
**18**	3.2121	113.5007	11	ND
**19**	3.2142	113.5035	12	ND
**20**	3.1559	113.3360	11	ND
**21**	3.1524	113.3277	11	ND
**22**	3.1661	113.3467	12	ND

Table S5: Plot locations, Sarawak, Malaysia. *Coordinates of OPs destructively harvested (D) and non-destructive plot surveys (ND), decimal degrees. Years after planting (YAP) at the time of measurement recorded (February 2019).*

Supplementary References

57) Melling L., Goh K.J., Beauvais C. & Hatano R. Carbon flow and budget in a young mature oil palm agroecosystem on deep tropical peat. In: Proc. Conf. International Symposium and Workshop on Tropical Peatland. International Peat Society, Yogyakarta, Indonesia, (2007).
58) Breure, C.J. Factors affecting yield and growth of oil palm tenera in West New Britain. *Oleagineux*, **37**, 213-227 (1982).
59) Breure, C.J. The effect of palm age and planting density on the partitioning of assimilates in oil palm (Elaeis guineensis). Exp. Agric. **24**, 53-66 (1988).
60) Dufrene E. (1989). Photosynthese, consommation en eau et modelisation de la production chez le palmier a huile (Elaeis guineensis Jacq.). (Universite Paris-Sud Orsay, 1989).
61) Henson, I.E. Carbon assimilation, water use and energy balance of an oil palm plantation assessed using micrometeorological techniques. In: Proc. Conf. 1993 PORIM International Palm Oil Congress. Palm Oil Research Institute of Malaysia, Kuala Lumpur (1995).
62) Lamade E and Setiyo I E. (1996). Test of Dufrene's production model on two contrasting families of oil palm in North Sumatra In: Proc. Conf. 1996 PORIM International Palm Oil Congress, Agricultural Conference. Palm Oil Research Institute of Malaysia, Kuala Lumpur (1996).
63) Henson I. E. Notes on oil palm productivity. I. Productivity at two contrasting sites. *J. Oil Palm Res.* **10**, 57-67 (1998).
64) Palm, C.A. et al. Carbon sequestration and trace gas emissions in slash-and-burn and alternative land uses in the Humid Tropics. (ACB Climate Change Working Group, Final Report Phase II, 1999).
65) Tjitrosemito, S. & Mawardi, I. Terrestrial carbon stock of oil palm plantation. In: Proc. Conf. Science and Policy Workshop on Terrestrial Carbon and Possible Trading under the CDM. IC-SEA, BIOTROP, Bogor, Indonesia, 2000.
66) Banabas, M. Agronomy Field Trial 305, Final Report. PNGOPRA, Papua New Guinea, 2002.
67) Henson I.E., Zuraidah, Y., Mohd Roslan, M.N., Mohd Haniff, H. & Tarmizi, A.M. Predicting soil water status, evapotranspiration, growth and yield of oil palm in a seasonally dry region of Malaysia. *J. Oil. Palm. Res.* **19**, 398-415 (2007).
68) Ng S. K., Thamboo, S. & de Souza, P. Nutrient contents of oil palms in Malaya. II. Nutrients in vegetative tissues. Malay. Agric. **46**, 332-390 (1968).
69) Legros, S. et al. (2006). Carbohydrate reserves in 9 years old oil palm: nature, distribution and seasonal changes. In: Proc. Conf. 2006 International Oil Palm Conference, Bali, 2006.
70) Koh, L.K., Rumpang, E., Kamarudin, N. and Haryn. M.H. Quantifying total carbon stock of mature oil palm. *J. Oil. Palm. Res.* **31**, 521-527 (2019).
71) PNGOPRA. *2011 Annual Research Report.* (Papua New Guinea Oil Palm Research Association, Kimbe, Papua New Guinea, 2011).
72) BLRS. *Annual Research report, 2001*. (Bah Lias Research Station, North Sumatra, 2002).
